# Skeletal Muscle Recovery from Disuse Atrophy: Protein Turnover Signaling and Strategies for Accelerating Muscle Regrowth

**DOI:** 10.3390/ijms21217940

**Published:** 2020-10-26

**Authors:** Timur M. Mirzoev

**Affiliations:** Myology Laboratory, Institute of Biomedical Problems RAS, Moscow 123007, Russia; tmirzoev@yandex.ru

**Keywords:** skeletal muscle, disuse atrophy, unloading, recovery, reloading, protein synthesis, protein degradation, muscle regrowth

## Abstract

Skeletal muscle fibers have a unique capacity to adjust their metabolism and phenotype in response to alternations in mechanical loading. Indeed, chronic mechanical loading leads to an increase in skeletal muscle mass, while prolonged mechanical unloading results in a significant decrease in muscle mass (muscle atrophy). The maintenance of skeletal muscle mass is dependent on the balance between rates of muscle protein synthesis and breakdown. While molecular mechanisms regulating protein synthesis during mechanical unloading have been relatively well studied, signaling events implicated in protein turnover during skeletal muscle recovery from unloading are poorly defined. A better understanding of the molecular events that underpin muscle mass recovery following disuse-induced atrophy is of significant importance for both clinical and space medicine. This review focuses on the molecular mechanisms that may be involved in the activation of protein synthesis and subsequent restoration of muscle mass after a period of mechanical unloading. In addition, the efficiency of strategies proposed to improve muscle protein gain during recovery is also discussed.

## 1. Introduction

Skeletal muscles play fundamental roles in the human body, including locomotion, posture maintenance, generating heat, venous blood flow, and breathing control. Furthermore, making up about 40–45% of the body’s mass, skeletal muscles also play a crucial role in the regulation of whole-body metabolism [1,2]. Accordingly, the maintenance of skeletal muscle mass and function is essential for mobility, disease prevention, and associated with overall health and quality of life [3,4,5]. Skeletal muscle tissue has a unique ability to alter its metabolism and the size of myofibers in response to changes in mechanical loading. Indeed, chronic mechanical loading leads to an increase in skeletal muscle mass and an enlargement of muscle fibers, while prolonged mechanical unloading results in a significant decrease in muscle mass and the cross-sectional area (CSA) of muscle fibers (muscle atrophy) [6,7]. The maintenance of skeletal muscle mass is dependent on the balance between the rates of muscle protein synthesis and protein degradation. Protein synthesis is controlled by the efficacy with which mRNA is translated into peptides (i.e., translational efficiency) and the amount of translational machinery (first of all, the number of ribosomes) per unit tissue (i.e., translational capacity) [8,9]. Muscle protein degradation is carried out via three main pathways: ubiquitin–proteasome, autophagy/lysosome and calpain-dependent [10,11].

The most important event in the process of skeletal muscle recovery from unloading is the upregulation of anabolic processes followed by an increase in muscle mass and subsequent recovery of muscle performance. In this regard, it is very important to understand the changes in the activity of key intracellular signaling pathways that regulate protein synthesis in skeletal muscle.

Muscles that experience atrophy during unloading are more susceptible to injury when they are reloaded or reweighted. Riley and colleagues demonstrated that hindlimb muscles of rats removed about 48 h following spaceflight/unloading exhibited sarcomeric disruptions, Z-line streaming, and an infiltration of inflammatory cells [12,13]. Since similar events have also been observed during muscle injury following unaccustomed or eccentric exercise [14], it is reasonable to assume that the same mechanisms can be involved.

Muscle fibers atrophied due to prolong spaceflight/mechanical unloading are structurally weaker and more susceptible to eccentric-like (lengthening) contraction-induced tearing of the contractile elements, sarcolemma, and associated connective tissue [12,13,15,16]. The severity of the damage appears to be directly correlated to the magnitude of the reloading workload. The observed alterations are reminiscent of those associated with delayed-onset muscle soreness in human muscles after unaccustomed strenuous exercise and in rodent muscles electrically stimulated to generate eccentric contractions [15,17]. Adaptation to the lower workload history of microgravity/unloading appears to render skeletal muscle more prone to structural failure when reloaded. This is partly explained by the relatively greater workload on the antigravity muscles (such as soleus or adductor longus muscles) because of severe fiber atrophy [16]. Indeed, 14-day unloading-induced loss of rat soleus muscle mass (about 50%) [18] is equivalent to increasing muscle loading by doubling the body weight.

The hypothesis about fundamental similarities between acutely reloaded skeletal muscle and skeletal muscle following a bout of eccentric contractions was confirmed by reports demonstrating that during early reloading in rat soleus muscle occurs both sarcolemmal disruptions [19] and an increased activity of calcium (Ca^2+^)-activated proteases (calpains) [20] resulting in a significant decrease in the content of cytoskeletal proteins [21]. On the other hand, it is known that after an eccentric load, there is a sharp activation of anabolic signaling in skeletal muscles fibers [22,23,24], therefore, it can be assumed that during the initial period of reloading, components of the mammalian/mechanistic target of rapamycin complex 1 (mTORC1) signaling system may be involved, leading to an increase in the rate of protein synthesis.

While molecular mechanisms regulating protein synthesis and degradation during mechanical unloading have been relatively well studied, signaling events implicated in protein turnover during skeletal muscle recovery from unloading are poorly defined. A better understanding of the molecular events that underpin muscle mass recovery following disuse-induced atrophy is of significant importance for both clinical and space medicine. This review focuses on the molecular mechanisms that may be involved in the activation of protein synthesis and subsequent restoration of muscle mass after a period of mechanical unloading. In addition, the efficiency of strategies proposed to improve muscle protein gain during recovery is also discussed.

## 2. Regulation of Protein Synthesis and Protein Degradation in Skeletal Muscle

Skeletal muscle protein synthesis and protein breakdown are regulated by an intricate network of signaling pathways that get activated or inactivated in response to various stimuli such as mechanical tension, nutrients, hormones/growth factors, etc. To date, different anabolic and catabolic signaling pathways in skeletal muscle have been uncovered and a lot of excellent recent reviews are available elsewhere in the literature [8,25,26,27,28,29,30,31]. Hence, only a brief overview of the mechanisms that control translational capacity and efficiency will be presented in the present section of the review. Since mechanical loading plays a key role in skeletal muscle adaptation to unloading and subsequent reloading, a role for mechanosensitive pathways regulating translational capacity (ribosome biogenesis) and efficiency in skeletal muscle will also be discussed.

### 2.1. Regulation of Ribosome Biogenesis

**Figure 1:**
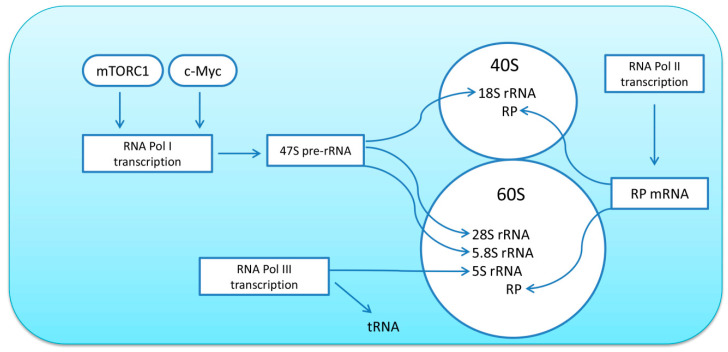
Simplified diagram showing the key regulatory factors involved in ribosome biogenesis. Arrows indicate stimulatory signals. mTORC1—mammalian/mechanistic target of rapamycin complex 1, c-Myc—c-myelocytomatosis oncogene, RNA Pol I, II or III—RNA polymerases I, II or III, rRNA—ribosomal RNA, tRNA—transfer RNA, RP—ribosomal proteins, 40S—small ribosomal subunit, 60S—large ribosomal subunit.

The ribosome is composed of one 40S and one 60S subunit. The 40S subunit includes 33 ribosomal proteins (RPs) and the 18S rRNA; while the 60S subunit consists of 46 RPs and the 5S, 5.8S, and 28S rRNAs [27]. The quantity of ribosomes is one of the key determinants of translational capacity within the cell; thus, alterations in ribosome biogenesis can promote or limit the rate of protein synthesis in skeletal muscle fibers. Ribosome biogenesis is a complex process that involves the activity of all three RNA polymerases: RNA polymerase I (Pol I) transcribes 47S pre-rRNA, a further processing which gives rise to the mature 18S, 5.8S, and 28S rRNAs; RNA polymerase II (Pol II) transcribes mRNAs (including genes encoding for ribosomal proteins, RP); RNA polymerase III (Pol III) transcribes 5S rRNA, tRNA, and other small RNAs (for review, see [8]) (Figure 1). Transcription of ribosomal DNA (rDNA) by Pol I is considered to be the rate-limiting step; however, de novo synthesis of ribosomes requires coordinated synthesis of equimolar amounts of all four types of rRNAs, as well as about 80 ribosomal proteins, and therefore involves all three polymerases [27].

Mammalian/mechanistic target of rapamycin complex 1 (mTORC1) and c-myelocytomatosis oncogene (c-Myc) are considered to be master regulators of ribosome biogenesis in skeletal muscle (see recent reviews [8,32]). Both molecules can promote transcription of the 47S pre-rRNA via activation of selective factor 1 (SL1) and upstream binding factor (UBF) that bind to the rDNA promoter and stabilize the initiation complex. mTORC1 associates with RNA Pol III genes through the binding with transcription factor IIIC, a DNA-binding factor that recognizes the promoters of these genes [9]. mTORC1 also promotes the translation of the RP and other accessory proteins through a 5′-TOP (5′-terminal oligopyrimidine tract) mechanism. Furthermore, von Walden et al. (2016) reported that mTOR associates with rDNA promoter in muscle cells and participates in chromatin remodeling leading to the activation of transcription of ribosomal genes [33]. These findings demonstrate that mTORC1, independent of its well-known function in the regulation of translational efficiency, is able to promote cell growth through direct regulation of ribosome biogenesis. Another key regulator of ribosome biogenesis, transcription factor c-Myc, is known to impact Pol I-mediated transcription of rRNA by binding to the promoter of rDNA, indirectly by regulating the expression and/or the recruitment of UBF and SL-1 and by promoting chromatin decondensation near rDNA loci via the acetylation of histones H3 and H4 [9]. Moreover, c-Myc can control the expression of auxiliary factors involved in rRNA processing, ribosome assembly, and nuclear ribosome export [9].

### 2.2. Mechanosensitive Pathways Regulating Translational Capacity and Efficiency in Skeletal Muscle

Myofibers are highly mechanically active and the amount of contractile activity and mechanical stimuli determines the size of myofibrils. Therefore, skeletal muscle fibers are equipped with special mechanosensory structures that can transform mechanical perturbations into molecular signals involved in the processes regulating protein synthesis (translational capacity and efficiency). In skeletal muscle, mechanosensory elements are mainly localized to the sarcolemma (for example, integrin-linked focal adhesion complexes, stretch-activated ion channels (SAC)), or sarcomere (a complex of titin domains and associated proteins).

**Figure 2:**
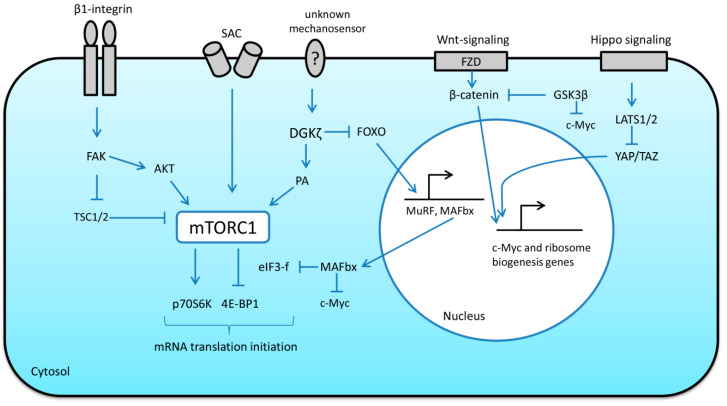
Simplified diagram depicting mechanoresponsive signaling pathways in skeletal muscle involved in the regulation of translational capacity and efficiency. Arrows indicate stimulatory signals, whereas T bars represent inhibitory signals. SAC—stretch-activated ion channels, FZD—frizzled protein (receptor), FAK—focal adhesion kinase, AKT—protein kinase B, TSC1/2—tuberous sclerosis complex1/2, DGKζ—zeta isoform of diacylglycerol kinase, PA—phosphatidic acid, mTORC1—mammalian/mechanistic target of rapamycin complex 1, p70S6K —ribosomal protein S6 kinase p70, 4E-BP1—eukaryotic initiation factor 4E binding protein, FOXO—forkhead box O protein, MuRF1—muscle RING finger, MAFbx—muscle atrophy F-box, c-Myc—c-myelocytomatosis oncogene, eIF3f—eukaryotic initiation factor 3f, GSK3β—glycogen synthase kinase-3β, LATS1/2—large tumor suppressor kinase 1/2, YAP—Yes-associated protein, TAZ—transcriptional coactivator with PDZ-binding motif.

Wnt/β-catenin signaling has been shown to be responsive to mechanical stretch as well as extracellular matrix stiffness, suggesting that mechanical stimuli can be involved in the regulation of this pathway [31]. Armstrong and Esser (2005) provided the first evidence that Wnt/β-catenin signaling pathway can induce the activation of growth-control genes (including c-Myc) during overload-induced hypertrophy in skeletal muscle (mouse plantaris muscle) [34]. These authors also demonstrated that the expression of beta-catenin is necessary for physiological growth of skeletal muscle in response to mechanical overload [35]. In canonical Wnt signaling, the binding of the Wnt protein to specific membrane receptors leads to phosphorylation and activation of the disheveled protein (Dvl) [17]. Dvl is able to phosphorylate and inhibit glycogen synthase 3β (GSK-3β), a negative regulator of β-catenin. Accumulation of β-catenin causes translocation of this protein to the nucleus and subsequent activation of c-Myc expression [9] (Figure 2). There is evidence that GSK-3β is also able to reduce ribosome biogenesis by direct c-Myc (Thr 58) phosphorylation, which leads to c-Myc ubiquitination and destruction by the proteasome [36,37] (Figure 2). Interestingly, Mei et al. (2015) have shown that E3 ubiquitin ligase muscle atrophy F-box (MAFbx/Atrogin-1) can also induce c-Myc degradation and phosphorylation of c-Myc at Thr-58 is dispensable for this process [38]. MAFbx/Atrogin-1 was also demonstrated to target eukaryotic initiation factor 3f (eIF3f) for ubiquitination and degradation by the proteasome [39]. This case represents a possible link between molecules involved in the regulation of protein degradation (MAFbx/Atrogin-1) and translational efficiency/protein synthesis (eIF3f) (Figure 2).

Yes-associated protein (YAP) and its paralog transcriptional coactivator with PDZ-binding motif (TAZ) are transcription factors downstream of the Hippo-signaling pathway. The activity of these two proteins is involved in determining organ size [31]. Phosphorylation of YAP by large tumor suppressor kinase (LATS)1/2 sequesters YAP to the cytoplasm, restricting its transcriptional activity [31]. YAP was shown to be implicated in the transduction of mechanical signals that regulate various processes including cellular growth [40]. Goodman et al. (2015) showed that synergist ablation increases both the expression and phosphorylation of YAP and that overexpression of YAP induces hypertrophy through an mTORC1-independent mechanism [40]. Watt et al. (2015) demonstrated that the hypertrophic effects of YAP are associated with an increase in the rates of protein synthesis [41]. Thus, increased YAP/TAZ expression stimulates protein synthesis and muscle fiber hypertrophy via mTORC1-independent mechanism [40,41] suggesting that the expression of YAP/TAZ is sufficient to alter transcription of genes involved in the regulation of protein synthesis. Indeed, evidence suggests that overexpression of YAP could be associated with an increase in c-Myc expression and, hence, enhanced translational capacity in skeletal muscle [40] (Figure 2).

Structures linking the muscle fiber membrane to the extracellular matrix, such as integrins and focal adhesion complex proteins, undergo conformational changes when force is applied to them, which can affect intracellular signaling events. In skeletal muscle cells, it was shown that disruption of proteins of the focal adhesion complex can blunt intracellular anabolic signaling [42]. Moreover, these focal adhesion complexes can directly activate ribosomal proteins to facilitate mRNA translation [43]. In skeletal muscle, focal adhesion kinase (FAK) can play an important role in the transmission of mechanical cues to mTORC1 signaling and protein synthesis [43,44] (Figure 2).

Mechanical deformations of the sarcolemma can also be sensed by SAC. The activity of these mechanosensitive channels was shown to be involved in the regulation of anabolic response to mechanical stimuli in the form of eccentric contractions. Pharmacological inhibition of SAC resulted in a significant downregulation of mTORC1 signaling (p70S6K Thr389 phosphorylation) in skeletal muscle in response to mechanical loading [45,46] (Figure 2).

mTORC1 signaling serves as a master controller of protein synthesis and cellular growth, integrating a number of upstream signals, including mechanical stimuli. mTORC1 plays a fundamental role in mechanically induced skeletal muscle protein synthesis and growth (for reviews, see [47,48,49,50]). Both increased and decreased mechanical loads were shown to impact mTORC1 signaling in mammalian skeletal muscle [51,52,53,54]. mTORC1 is known to be implicated in both translational efficiency and capacity by regulating all three polymerases [55] and is required for an acute increase in muscle protein synthesis in response to mechanical cues [56,57,58], whereas prolonged protein synthesis in skeletal muscle may occur via mTORC1-independent mechanisms [59].

Mechanical load-induced mTORC1 activation and subsequent skeletal muscle hypertrophy can be inhibited by specific inhibitors, such as rapamycin [56]. The exact molecular mechanisms which could be involved in mTORC1 activation in response to mechanical stimuli are vaguely defined; nonetheless, evidence suggests that diacylglycerol kinase ζ (DGKζ)-mediated production of phosphatidic acid (PA) can play a key role in this process [60]. Interestingly, DGKζ has been recently shown to inhibit muscle proteolysis via the forkhead box protein O (FoxO)-dependent pathway [61], thereby providing another link between anabolic and catabolic signaling pathways (Figure 2). Moreover, subcellular localization of mTORC1 may play an important role in mechanically induced mTORC1 activation. Under resting conditions, skeletal muscle lysosomes are enriched with PA, mTOR and tuberous sclerosis complex 2 (TSC2) (endogenous inhibitor of mTORC1). The presence of TSC2 on the lysosomes keeps mTORC1 signaling in a relatively inactive state [62]. Eccentric muscle contractions induce phosphorylation of TSC2, causing it to dissociate from the lysosomal surface, thereby promoting the activation of mTORC1 signaling [62].

**Figure 3:**
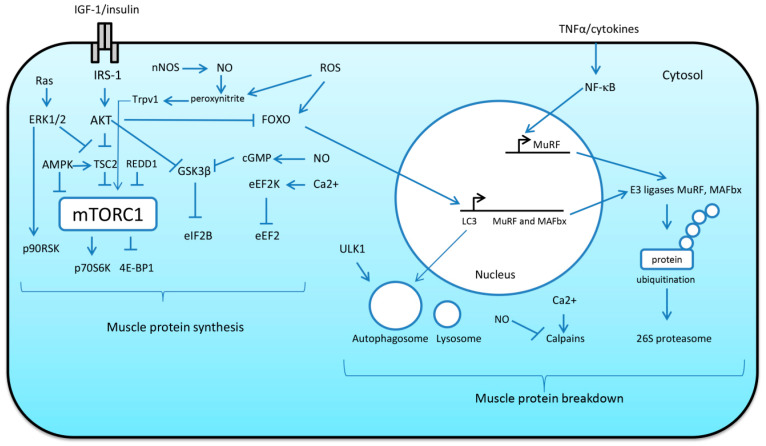
Simplified diagram delineating key signaling pathways involved in skeletal muscle protein synthesis and degradation. Arrows indicate stimulatory signals, whereas T bars represent inhibitory signals. IGF-1—insulin-like growth factor 1, TNFα—tumor necrosis factor alpha, ERK1/2—extracellular signal-regulated kinase 1/2, IRS-1—insulin receptor substrate 1, AMPK—AMP-activated protein kinase, AKT—protein kinase B, TSC1/2—tuberous sclerosis complex1/2, NO—nitric oxide, nNOS—neuronal NO synthase, Trpv1—transient receptor potential cation channel subfamily V member 1, ROS—reactive oxygen species, REDD1—regulated in development and DNA damage response 1, mTORC1—mammalian/mechanistic target of rapamycin complex 1, p70S6K—ribosomal protein S6 kinase p70, 4E-BP1—eukaryotic initiation factor 4E binding protein, FOXO—forkhead box O protein, MuRF1—muscle RING finger, MAFbx—muscle atrophy F-box, c-Myc—c-myelocytomatosis oncogene, eIF3f—eukaryotic initiation factor 3f, GSK3β—glycogen synthase kinase 3β, eIF2B—eukaryotic initiation factor 2B, cGMP—cyclic guanosine monophosphate, eEF2K—eukaryotic elongation factor 2 kinase, eEF2—eukaryotic elongation factor 2, p90RSK—ribosomal protein S6 kinase p90, ULK1—unc-51-like autophagy activating kinase, LC3—microtubule-associated proteins 1A/1B light chain 3B, NF-κB—nuclear factor κB.

A possible role of nitric oxide (NO) in the regulation of skeletal muscle mass was demonstrated when inhibition of NO synthase (NOS) by NG-nitro-L-arginine methyl ester (L-NAME) administration significantly attenuated muscle hypertrophy induced by mechanical overload of rat skeletal muscle [63,64]. Furthermore, Ito and co-workers (2013) revealed a signaling pathway by which neuronal NOS (nNOS)-mediated NO production could mediate muscle hypertrophy [65]. Upon mechanical loading, nNOS derived NO can react with superoxide to generate peroxynitrite. Peroxynitrite is able to activate transient receptor potential cation channels, subfamily V, member 1 (TRPV1) causing an increase in intracellular Ca^2+^ levels via release from the sarcoplasmic reticulum [65]. Elevations in intracellular Ca^2+^ lead to TORC1 activation and increased protein synthesis via yet unknown mechanism (Figure 3). In support of this mechanism of mTORC1 activation, it was also shown that administration of the TRPV1 agonist capsaicin activated TORC1 signaling to a comparable level as that observed with mechanical overload [65]. The role of NO signaling in skeletal muscle growth was uncovered by De Palma et al. (2014) who showed that in a mouse model in which nNOSμ is absent, impaired muscle growth during development via an upregulation of proteolytic pathways occurs [66]. Interestingly, NO may also be implicated in phosphorylation and inhibition of GSK-3β (a negative regulator of protein synthesis) via cyclic guanosine monophosphate (cGMP)-dependent protein kinases [67].

### 2.3. Roles of IGF-1/AKT, MAPK/ERK Pathways and NF-κB Signaling in the Regulation of Translational Efficiency and Protein Degradation

It is well-known that activation of the phosphotidylinositol-3-kinase (PI3K)/protein kinase B (AKT) pathway by insulin-like growth factor 1 (IGF-1) can lead to increases in protein synthesis via AKT-mediated phosphorylation of TSC2, an endogenous inhibitor of TORC1 [68,69]. AKT-dependent phosphorylation of TSC2 inhibits its activity, resulting in the accumulation of the active form of Ras homolog enriched in brain (Rheb), a potent TORC1 activator. Activated TORC1 promotes protein synthesis by phosphorylating ribosomal protein S6 kinase (p70S6K) and eukaryotic initiation factor 4E binding protein (4E-BP1), two factors that enhance the initiation of mRNA translation (Figure 3). Another downstream target of PI3K/AKT signaling is GSK-3β, a negative regulator of protein synthesis [70]. AKT-mediated phosphorylation of GSK3β results in its inactivation, which prevents inhibition of mRNA translation initiation via eukaryotic initiation factor 2Bε (eIF2Bε) [70] and thereby contributes to translational efficiency (Figure 3). Moreover, IGF-1-dependent activation of AKT results in the repression of MAFbx and muscle RING finger protein 1 (MuRF-1) expression via FoxO phosphorylation [25] (Figure 3). It was shown that overexpression of a constitutively active form of FoxO3 isoform caused a significant increase in MAFbx expression and reduced myotube diameter by 50% [71]. Both E3 ubiquitin ligases MAFbx and MuRF-1 are muscle-specific components of the ubiquitin–proteasome system (UPS) which is responsible for degrading most individual proteins [25]. E3 ubiquitin ligases catalyze the rate-limiting step in ubiquitin conjugation and are substrate-specific, providing selectivity to the UPS. Proteins tagged with ubiquitin are then imported to the 26S proteasome for digestion [25].

Another important proteolytic system in skeletal muscle is the calpain system (interested readers are referred to an excellent review by Hyatt and Powers (2020) on the role of calpains in skeletal muscle plasticity [72]). Calpains are Ca^2+^-activated non-lysosomal proteases involved in cleavage of target proteins [73]. The two primary calpains that contribute to skeletal muscle atrophy are calpain 1 and calpain 2 [74]. Activated calpains are reported to cleave more than 100 different proteins including such cytoskeletal proteins as titin and nebulin [74]. Of note, oxidation of contractile proteins (actin, myosin) can increase their susceptibility for degradation by calpains [75]. It is important to note that inhibition of calpains can protect skeletal muscles of rodents against disuse-induced muscle atrophy [76,77]. Apart from calpain 1 and 2, it was shown that calpain 3 may participate in sarcomere remodeling by acting upstream of the ubiquitin–proteasome pathway [78]. It is also worth noting that in skeletal muscle cells, it was demonstrated that NO is able to inhibit m-calpain activity and cytoskeletal proteolysis [79].

Transcription factor FoxO3 is also involved in regulation of autophagy. Autophagy is a mechanism of protein breakdown that utilizes autophagosomes and lysosomes to facilitate degradation and recycling of cellular components [25]. During autophagy, dysfunctional organelles and protein aggregates are sequestered into double membrane vesicles called autophagosomes. Then, these autophagosomes fuse with lysosomes to form autolysosomes [80]. Following fusion with the lysosome, the contents of the autophagosomes are degraded by lysosomal proteases (i.e., cathepsins) [80]. Unc-51-like autophagy activating kinase (ULK1) plays an important role in the initiation of autophagy process (Figure 3). The activity of ULK1 is negatively regulated by mTORC1 and positively regulated by AMP-activated protein kinase (AMPK) [81]. AMPK is an energy sensor that plays an important role in cell metabolism, and protein synthesis in particular. It was shown that AMPK can inhibit protein synthesis through phosphorylation of TSC2 (TORC1 inhibitor) [82], as well as via phosphorylation of Raptor (regulatory-associated protein of mTOR) [83]. Another signaling molecule, protein regulated in development and DNA damage 1 (REDD1), has also been shown to inhibit mTORC1 signaling in skeletal muscle (for a comprehensive review, see [84]). AMPK has also been shown to promote FoxO expression and subsequent induction of MAFbx and MuRF-1 [85]. In skeletal muscle, FoxO3 directly controls autophagy through the transcription of autophagy-related genes [86]. Although autophagy is a proteolytic process, and excessive autophagy is known to contribute to atrophy under catabolic states [87], inhibition of autophagy can result in atrophy and myopathy and is required to maintain muscle mass [88].

Apart from translation initiation, translational efficiency also depends upon the process of mRNA translation elongation. Eukaryotic elongation factor 2 (eEF2) is known to be a key regulator of polypeptide chain elongation that catalyzes translocation of the ribosome [89]. Phosphorylation of eEF2 at Thr56 results in the inhibition of mRNA translation elongation [90]. This inhibitor phosphorylation is mediated by eukaryotic elongation factor 2 kinase (eEF2K) [90] (Figure 3). In turn, eEF2K may be subject to inhibiting or activating phosphorylation via various regulatory mechanisms. For instance, p70S6K and ribosomal S6 kinase p90 (p90RSK) can phosphorylate (by Ser366 and Ser359) and inhibit eEF2K activity, leading to increased protein synthesis [91,92]. At the same time, an increased calcium concentration as well as phosphorylation by protein kinase A (PKA) and AMPK can lead to eEF2K activation and subsequent inhibition of translation elongation [92,93,94].

Another important biochemical cascade involved in both mTORC1-dependent and mTORC1-independent regulation of protein synthesis is the Ras/ERK/p90RSK signaling pathway (Figure 3). From the literature, it is known that ERK and p90RSK kinases can phosphorylate and inhibit TSC2 protein, an inhibitor of mTORC1 [95,96]. ERK and p90RSK can also directly activate mTORC1 by phosphorylating the Raptor protein [97]. In addition, p90RSK can participate in mTORC1-independent activation of translation by phosphorylating regulatory proteins such as rpS6 [95] and eEF2K [98].

NF-κB is a transcription factor implicated in a range of biological processes including inflammatory and immune responses and is rapidly activated by inflammatory cytokines such as TNF-α (for review see [99]) (Figure 3). It was demonstrated that NF-κB is able directly to regulate the expression of MuRF-1 via a Bcl-3 dependent mechanism [100]. Moreover, Wu et al. (2014) revealed that NF-κB sites are required for MuRF-1 promoter activation in rat soleus muscle during mechanical unloading [101]. Furthermore, in C2C12 myotubes, it was shown that NF-κB is essential for TWEAK (TNF-like weak inducer of apoptosis)-induced expression of MuRF1 and Beclin-1 [102].

To summarize, in the last few decades, significant advancements have been made in our understanding of the anabolic and catabolic signaling pathways implicated in the regulation of skeletal muscle mass. Since skeletal muscle is susceptible to alternations in mechanical load, mechanical stimuli are able to elicit changes in both translational efficiency and translational capacity via changes in mechanosensitive pathways. While there still remains much to be learned, one conclusion that is clear is that the regulation of skeletal muscle mass during periods of increased or decreased mechanical loading represents a complex crosstalk between multiple signaling pathways regulating protein synthesis and proteolysis.

## 3. Effects of Reloading on Skeletal Muscle Mass, Protein Synthesis and Protein Turnover Signaling

### 3.1. Effect of Reloading on Muscle Mass and Fiber Size

Recovery of wet skeletal muscle mass is normally complete after 14 days of reloading following 14-day mechanical unloading [103,104,105,106], whereas processes related to fiber CSA recovery after prolonged hindlimb unloading (HU) can extend up to 5 weeks [107]. Musacchia et al. (1990) showed that it takes a week to restore wet mass of rat soleus muscle after 7-day HU [108]. It is important to note that despite a fairly intensive recovery of wet muscle mass during the first week of the reloading period [109], an increase in dry muscle weight is relatively small [110,111]. This may indicate that an increase in muscle mass during the first days of reloading may be attributable to interstitial fluid accumulation (edema) and do not actually represent an increase in protein mass [112]. This phenomenon can explain the discrepancy between changes in muscle mass and fiber CSA in the course of reloading (the recovery of myofiber CSA in atrophied muscle takes longer than muscle mass recovery) [103,107,113,114].

### 3.2. Effect of Reloading on Muscle Protein Synthesis

Tucker et al. (1981) were the first who assessed the rates of protein synthesis in rat gastrocnemius muscle during recovery from 7-day hindlimb immobilization [115]. They revealed that fractional rates of protein synthesis significantly increased the control values during the first 6 h of recovery, then remained at control values for the next 2 days, and finally significantly increased to about twice the control values on the 4th day of recovery from disuse [115]. Taillandier et al. (2003) showed that, following 18 h of reloading after 9-day HU, protein synthesis in rat soleus muscle was increased by 65% vs. control [116]. Furthermore, protein synthesis appeared to be elevated after 7 days of reloading (+76%) compared to control [116]. These data are consistent with subsequent studies in which a significant increase in the rate of protein synthesis in rat soleus muscle at various stages of reloading was shown [104,105,117] (Table 1).

### 3.3. Signaling Pathways Involved in the Regulation of Protein Synthesis during Muscle Reloading

It is well known that protein synthesis can be regulated by IGF-1 signaling. Litvinova and co-authors (2007) showed that on the 3rd day of reloading after 14-day unloading, IGF-1 serum concentration in rats was significantly lower compared to control values [110]. At the same time, on the 7th day of reloading, a sharp increase in the concentration of circulating IGF-1 relative to the control level was observed [110]. However, on the 7th day of reloading after prolonged HU (28 days), IGF-1 content in rat soleus muscle remained reduced and completely recovered by 28-day reloading [118]. Reduced IGF-1 content in the soleus muscle after 7-day reloading could be associated with a decrease in IGF-1 mRNA expression as well as an increased secretion of IGF-1 into intercellular space (autocrine regulation). Kachaeva and co-authors (2010) demonstrated that IGF-1 mRNA expression in soleus muscle of male Wistar rats after 3-day reloading did not differ from control, however, a sharp increase in IGF-1 mRNA expression was observed after 7-day recovery from unloading [111]. On the other hand, Heinemeier et al. (2009) showed a significant increase in the mRNA expression of IGF-1 as well as MGF (mechano-dependent growth factor) and TGF-β1 (transforming growth factor beta-1) in soleus muscle of female Sprague-Dawley rats after 2, 4, and 8 days of reloading relative to control values [119]. By the 16th day of recovery period, the expression of these growth factors did not differ from the level of control [119]. A significant increase in the expression of IGF-1 mRNA was observed in the plantar and soleus muscles of mice following 3-day reloading after two weeks of HU [106]. Of note, the increase in IGF-1 expression after 3-day reloading was more pronounced in the soleus muscle as compared to the plantaris muscle [106]. After 5, 7, and 14 days of reloading, the expression of IGF-1 in the mouse soleus did not differ from control values, whereas in mouse plantaris a spike in the expression of this growth factor was also observed on the 7th day of the recovery period [106]. It is also important to note that during acute reloading the pattern of expression of IGF-1 isoforms in skeletal muscle is generally similar to that observed after eccentric loading [120,121].

Stevens-Lapsley et al. (2010) previously evaluated the effect of viral-mediated IGF-I overexpression on muscle size and function during recovery after a period of cast immobilization in fast-twitch muscles [122]. Relative gains in both wet weight and fiber size during 3-week reloading were significantly larger in the IGF-I- injected vs. phosphate-buffered saline (PBS)-injected extensor digitorum longus muscles [122]. This finding is in line with a study by Ye et al. (2013) which demonstrated that IGF-1 overexpression attenuated reloading-induced muscle damage in murine soleus muscle, and accelerated muscle regeneration and force recovery [123].

Possible role of NO in the activation of mTOR and muscle regrowth during recovery from disuse atrophy was recently studied by Aguiar and co-workers (2017) [124]. Using pharmacological inhibitors of NO production (1-(2-trifluoromethyl-phenyl)-imidazole (TRIM) and L-NAME) during 7-day recovery from 10-day hindlimb immobilization, the authors discovered that the recovered group displayed a complete plantaris muscle regrowth compared to control group, but the TRIM and L-NAME groups remained atrophied [124]. In addition, there was a 29% increase in phospho-mTOR (Ser2448) protein expression in the recovered group relative to control group, and this increase was blocked in both TRIM and L-NAME groups [124]. Thus, NO appears to be an important molecule for skeletal muscle regrowth following immobilization.

Kawada et al. (2001) showed that the content of myostatin, a negative regulator of protein synthesis, in mouse soleus muscle did not change after 14-day HU, but significantly decreased after a 2-day recovery period [125].

Taking into account that the effect of acute reloading on skeletal muscle is essentially similar to that seen after eccentric contractions, the activation of the key AKT/mTORC1/p70S6K signaling pathway should be expected during the first hours or days of muscle recovery after mechanical unloading. The importance of this signaling pathway in skeletal muscle recovery after a period of disuse was demonstrated by Bodine et al. (2001) [56]. The use of rapamycin (TORC1 inhibitor) significantly reduced the growth of skeletal muscle mass in rodents during the first week of recovery after HU [56]. The important role of mTOR in restoring protein synthesis and muscle mass of atrophied skeletal muscle was shown in an elegant experiment by Lang et al. (2012), in which mTOR heterozygous (mTOR ^(+/−)^) mice were used [126]. In such heterozygous mice, the content of mTOR in various tissues, including skeletal muscles, is reduced by about 50%. It turned out that the recovery of gastrocnemius muscle mass after immobilization in heterozygous mice was significantly slower compared to normal animals [126]. The lack of complete recovery of the immobilized limb mass in mTOR heterozygous mice was accompanied by a reduced rate of protein synthesis, a decrease in 4E-BP1 phosphorylation, and a decrease in the content of Raptor-4E-BP1 and eIF4G-eIF4E complexes [126]. In addition, unlike wild-type mice, mTOR heterozygous mice did not show an increase in IGF-1 mRNA expression in gastrocnemius muscle after 3 and 10 days of reloading [126].

A recent report suggested that both AKT-dependent and AKT-independent signaling pathways can contribute to the activation of protein synthesis in rat soleus muscle during 3-day reloading after HU [127]. The use of an inhibitor of phosphotidylinositol-3-kinase (PI3K) during 3-day reloading resulted in attenuation of both AKT (Ser473) phosphorylation and protein synthesis, and the use of an inhibitor of PA production led to a significant decrease in both p70S6K (Th389) phosphorylation and the rate of protein synthesis [127]. Thus, both PI3K/AKT-dependent and AKT-independent (possibly PA-dependent) pathways may be involved in the protein synthesis activation in rat postural muscle at the early stage of recovery from disuse-induced atrophy.

A possible role of AMPK, an endogenous mTORC1 inhibitor, in skeletal muscle mass recovery after a period of unloading was studied by Egawa et al. (2018) [128]. There was no difference in the regrowth of soleus muscle mass between wild-type mice and skeletal-muscle-specific dominant-negative AMPKα1 (AMPK-DN) mice after 7 days of reloading; however, by the 14th day of recovery, muscle regrowth was significantly higher in AMPK-DN mice [128].

Pansters et al. (2015) elucidated a role of another negative regulator of protein synthesis, GSK-3β, during reloading of mouse skeletal muscle [129]. Using mice lacking muscle GSK-3β (GSK-3β KO), the authors tested a hypothesis that muscle mass recovery following mechanical unloading would be accelerated in the absence of GSK-3β [129]. Reloading-associated changes in muscle protein turnover were not affected by the absence of GSK-3β; however, soleus muscle mass and fiber CSA regain in GSK-3β KO mice were enhanced compared to wild-type mice after 5-day reloading [129]. Using constitutively active Ser21/9 GSK-3α/β knock-in mice, the same group of authors have recently reported that phosphorylation of Ser-mediated GSK-3 inactivation is not required for reloading-induced muscle mass recovery [113]. Thus, these findings suggest that although GSK-3β activity can suppress soleus mass recovery after disuse atrophy, suppressive actions of GSK-3β do not appear to be regulated by Ser9 phosphorylation [113].

During the first days of reloading, an increase in circulating IGF-1 is not observed [110], however, as described above, the AKT/mTORC1 signaling pathway is activated and protein synthesis is enhanced. It can be associated either with autocrine IGF-1 regulation or mechanosensitive PI3K/AKT-independent signaling mechanisms [130]. Since mechanosensitive channels were shown to be involved in the activation of mTORC1 signaling after eccentric contractions [45] it can be assumed that mechanosensitive ion channels would play an important role in the activation of mTORC1 signaling and protein synthesis in the acute period of reloading. Indeed, it has been recently reported that functional stretched-activated channels are necessary for complete activation of mTORC1 signaling and protein synthesis in rat soleus muscle during an acute reloading (12h) after HU [117]. There is evidence that transient receptor potential canonical (TRPC) ion channels are likely molecular candidates for stretched-activated channels [131,132]. However, it is a debatable point since it was demonstrated that, under physiological conditions, TRPC1 channel may not exhibit mechanosensitive properties [132,133]. Nonetheless, Zhang et al. (2014) showed that TRPC1 protein expression is significantly decreased during the course of soleus muscle recovery (3, 7 and 14 days) from HU [103]. Furthermore, Xia and co-workers (2016) investigated the role of TRPC1 in the regulation of muscle regrowth after a period of mechanical unloading [114]. Microinjecting small interfering RNA (siRNA) into mouse soleus muscles after 7 days of reloading provided evidence for the role of TRPC1 in regulating muscle regrowth. The percentage of slow myosin heavy chain-positive myofibers, as well as myofiber CSA, was significantly lower in TRPC1-siRNA-expressing muscles than in control muscles after 14 days of reloading [114].

During the first days of reloading after immobilization or HU, an increase in phosphorylation of signal molecules such as AKT, GSK-3β, p70S6K, or rpS6 was observed in rat soleus muscle [127,134,135]. In addition, Baehr et al. (2016) showed that adult 9-month-old Fischer 344 rats after 3-day reloading showed a significant increase in phosphorylation of GSK-3β, p70S6K, 4E-BP1 in soleus and GSK-3β and p70S6K in tibialis anterior [105]. By 14 days of reloading after mechanical unloading, the content of phosphorylated forms of AKT, GSK-3β and p70S6K in the soleus of 6-month-old Fischer 344 rats did not significantly differ from the control values [136]. A recent study showed that an acute reloading (12 h) following HU was accompanied by a significant increase in the phosphorylation level of mTORC1-dependent molecules p-p70S6K (Thr389), p-rpS6 (Ser240/244) and p-4E-BP1 (Thr36/46) [117]. Choi et al. (2005) demonstrated that during the recovery period after HU, an activation of MEK/ERK1/2 signaling pathway in rats soleus muscle occurred [137]. Increased phosphorylation of AKT (Ser473) and GSK-3β (Ser9) in murine soleus muscle was observed after 3-day reloading after 14-day HU [106]. Increased phosphorylation (Ser9) and kinase activity of GSK-3β in murine soleus were also observed following 5-day reloading [106]. In addition, Ohno et al. (2014) showed a significant increase in the content of phosphorylated forms of AKT and p70S6K in mouse soleus muscle after 1- and 3-day reloading [138].

As for the activation of ribosomal biogenesis during reloading after HU, Heinemeier et al. (2009) showed that the total RNA content in soleus muscle of young female Sprague-Dawley rats was significantly higher than the control values following 2 and 4 days of recovery [119]. However, in adult 9-month-old male Fischer 344 rats, total RNA content in the soleus muscle during reloading did not differ from the control but was reduced after 14-day HU [105]. In addition, the expression of c-Myc mRNA in these adult rats did not significantly differ from the control either after 14-day unloading or during recovery period [105]. However, a recent report revealed a significant increase in c-Myc mRNA expression in soleus muscle of relatively young male Wistar rats during the course of acute reloading (6-, 12-, 24 h) after 14-day HU [117].

The above-described changes in the key intracellular anabolic markers in rodent soleus muscle during reloading are summarized in Table 1.

### 3.4. Regulation of Protein Degradation during Muscle Reloading after Unloading

Taillandier and colleagues (2003) demonstrated for the first time that total protein degradation in rat soleus was 22% higher (vs. control values) after 18 h of reloading and then returned to baseline by 7-day recovery [116]. It appears that an increase in protein breakdown in rodent skeletal muscles in the first few days after mechanical unloading is associated with the activation of the ubiquitin–proteasome degradation of proteins. This is evidenced by data on an increase in the expression of ubiquitin [116,139], the content of ubiquitin (Ub)-protein conjugates [78], and the expression of E3 ubiquitin ligases [111]. Moreover, during early reloading (18 h), but not at later stages (7 days), a significant increase in mRNA levels for C8 and C9 20S proteasome subunits in rat soleus muscle occurred [116]. Baehr et al. (2016) have measured ubiquitin levels and the activities of the three catalytic subunits (β1, β2, and β5) of the proteasome in rat skeletal muscles in response to reloading [105]. In the soleus muscle of adult rats (9 month) proteasome activity was significantly elevated at day 3 of reloading; however, in tibialis anterior muscle, proteasome activity during reloading did not change from baseline values [105]. Total ubiquitin levels in the soleus, but not the tibialis anterior, were also significantly increased following 3-day recovery from disuse atrophy [105]. In a recent study by Seaborne and co-authors (2019) a possible role of a novel E3 ubiquitin ligase UBR5 in skeletal muscle remodeling was demonstrated. From day 3 to day 14 of reloading after mechanical unloading, a linear increase in UBR5 protein abundance in rat gastrocnemius muscle occurred [140]. Interestingly, UBR5 protein levels were increased after 3-day HU but returned to baseline by 14-day HU [140]. Further research is necessary to determine the post-transcriptional regulation of UBR5 and its substrates in order to understand the role of this novel E3 ubiquitin ligase in skeletal muscle remodeling during disuse atrophy/recovery [140].

It has recently been shown that in the skeletal muscle of middle-age men, leg-immobilization-induced increase in RNA expression of E3 ubiquitin ligases (MuRF1 and MAFbx) returns to control levels by 14-day recovery [141].

The level of m-calpain mRNA expression was upregulated (+135%) in rat soleus following 18-h reloading and returned to baseline levels after 7-day reloading [116]. Enns and Belcastro (2006) have demonstrated that total calpain activities were upregulated during early reloading compared to both control conditions (for rat soleus and gastrocnemius) and HU only (rat gastrocnemius) [20]. Activation of calpains in this case could be associated with a significant increase in resting free cytosolic Ca^2+^ concentration that was previously demonstrated in mouse soleus muscle after 24-h reloading [142].

Using transgenic mice, Kramerova and colleagues demonstrated a role for muscle-specific calpain-3 during skeletal muscle recovery from unloading [78]. Calpain-3 knockout mice showed attenuated soleus muscle fiber growth during 2 and 4 days of reloading after HU. Unlike wild-type animals, during reloading soleus muscles from calpain-3 knockout mice did not accumulate Ub-protein conjugates. The results of that study suggest that calpain-3 and the UPS may act in series. Attenuated muscle recovery in the absence of calpain-3 could be associated with decreased protein turnover and accumulation of damaged or misfolded proteins [78]. It is well-known that UPS can prevent the accumulation of such non-functional proteins thereby facilitating cellular homeostasis [143]. Recently, it also has been shown that, apart from calpain-3, calcium calmodulin kinase IIβ signaling may be required to induce 70 kDa heat shock protein (HSP70) necessary for muscle regrowth following disuse [144]. Kneppers et al. (2019) have recently conducted a comprehensive analysis of autophagy markers in mouse gastrocnemius muscle during the course of reloading after 14-day HU [145]. The authors showed an acute but transient increase in the protein expression of the autophagosomes formation markers Map1lc3b-I, Gabarapl1, and Sqstm1 [145]. Further, the content of autophagy-related protein Beclin-1 was significantly increased (+230%) in rat soleus muscle after 5-day reloading compared to control values, suggesting autophagy activation [109].

In the early period of reloading a significant increase in the protein content of proinflammatory cytokines such as tumor necrosis factor alpha (TNFα) (1 and 5 days of reloading), interleukin-6 (IL-6) and interleukin-1β (1 day of reloading) was shown in the soleus muscle of female Wistar rats [109]. These cytokines are known to mediate proteolysis and muscle atrophy via NF-κB. Proinflammatory cytokines can be secreted by activated monocytes and macrophages. Evidence suggests that during early reloading, skeletal muscle is initially invaded by a phagocytic population of macrophages implicated in the degradation of the contents of injured muscle fibers. Peak concentrations of this population of macrophages are observed following 2 days of reloading [146]. However, after 4 days of skeletal muscle reloading, a second non-phagocytic population of macrophages reaches peak concentrations [146]. This non-phagocytic population is mostly distributed near regenerative fibers and can play an important role in regeneration of skeletal muscle after disuse [146]. Tidball and Wehling-Henricks (2007) reported that, between 2 and 4 days of reloading, the non-phagocytic macrophages contribute to mouse soleus muscle repair, growth, and regeneration [147]. In a subsequent study by Dumont and Frenette (2010), mice depleted in macrophages were submitted to HU and subsequent recovery to examine the roles of macrophages in muscle atrophy and regrowth. It was demonstrated that, during the early phase of reloading (1 and 3 days), macrophages neither prevent the loss in soleus muscle force nor promote recovery, however, they play a key role in soleus muscle growth and recovery following 7 and 14 days of reloading [148]. Furthermore, Washington et al. (2011) demonstrated the importance of IL-6 expression during the recovery of mouse gastrocnemius muscle from HU [149]. Following 1-day reloading, IGF-1 mRNA expression and Akt/mTOR signaling were upregulated in wild-type mice in WT muscle but attenuated in IL-6 knockout mice [149]. Moreover, IL-6 knockout mice showed a delayed restoration of the gastrocnemius muscle mass during 7-day reloading [149]. Thus, inflammatory/immune response appears to be an essential event at the early stage of skeletal muscle recovery from disuse-induced atrophy.

Alterations in the markers of proteolysis and inflammation in rodent soleus muscle during early reloading are summarized in Table 2.

To conclude, skeletal muscle recovery from disuse-induced atrophy is accompanied by the stimulation of both processes of protein turnover (protein synthesis and protein breakdown) which results in simultaneous destruction and synthesis of muscle proteins. Activation of proteolytic systems may be required to eliminate or replace damaged or atrophy-induced proteins while activation of protein synthesis contributes to rebuilding new proteins. It was shown that new protein isoforms representing up to 35% of total myofibrillar proteins appear during mechanical unloading and have to be replaced during reloading [150,151]. On the whole, in the course of reloading the rate of protein synthesis presumably prevails over the rate of proteolysis, leading to a complete recovery of skeletal muscle mass from disuse-induced atrophy.

## 4. Possible Strategies for Enhancing Skeletal Muscle Regrowth Following Disuse

### 4.1. Voluntary Wheel Running

A challenge during rehabilitation is to optimize strategies that elevate muscle recovery but minimize muscle injury. There is evidence that low-impact exercise, such as voluntary wheel running, may be useful during recovery of rodent skeletal muscles [152,153,154]. Ishihara et al. (2004) demonstrated that voluntary running exercise for 2 weeks during reloading significantly improved HU-induced changes in fiber CSA and fiber-type distribution in rat soleus muscle [152]. Hanson et al. (2010) showed that 7 days of muscle reloading with access to wheel-running can stimulate mouse soleus muscle to regain mass and fiber CSA [153]. Furthermore, Brooks et al. (2018) revealed that voluntary wheel running for 14 days during reloading after HU can effectively improve the recovery of mouse gastrocnemius muscle function and morphology [154]. The authors of that paper suggest that voluntary wheel running enhanced satellite cell proliferation during reloading after HU and this was linked to improved recovery and suppressed YAP (Ser127) phosphorylation levels compared to passive reloading (no wheel running) [154]. Future investigations will be needed to find out if YAP signaling is essential for wheel running-induced signaling of satellite cell proliferation or if other cofactors such as TAZ can replace YAP in this pathway [154].

In contrast to wheel running, forced exercise (e.g., treadmill running) may induce additional muscle damage leading to attenuated recovery after HU [155]. Bigard et al. (1997) also demonstrated that a more extensive muscle injury is observed when a 4-week endurance training running program is implemented during the recovery period from 21-day unloading [156]. Of note, Song et al. (2018) have recently shown that progressive resistance exercise (ladder climbing) during 4-week recovery from HU can promote an increase in rat hindlimb muscles strength without a significant increase in muscle weight [157].

### 4.2. Neuromuscular Electrical Stimulation

In situations when an increase in voluntary physical activity is not possible due to limb immobilization or general weakness in bedridden patients, neuromuscular electrical stimulation (NMES) can be a useful instrument to mimic exercise by inducing electrically evoked involuntary muscle contractions [158]. Indeed, in a clinical setting, NMES can be seen as an effective therapeutic approach to attenuate or prevent disuse-induced skeletal muscle atrophy in humans due to illness, injury or surgery [158,159]. Moreover, existing data suggest that the application of NMES during spaceflight is able to alleviate muscular atrophy in astronauts [159]. It is probable that beneficial effects of NMES under these conditions are associated with an enhanced rate of muscle protein synthesis and in some cases (5-day leg immobilization) may also be attributed to a suppression of proteolytic pathways [159]. In particular, Wall et al. (2102) observed trends for mTOR, p70S6K, and rpS6 phosphorylation to increase in human skeletal muscle immediately after 60 min of NMES and this increase in mTORC1 signaling appears to persist at 2 and/or 4 h following NMES [160]. Leg immobilization-induced upregulation in MAFbx, MuRF1 and myostatin mRNA expression in human skeletal muscle was prevented by NMES (40-min sessions, twice daily during 5-day immobilization) [161].

In rodent studies, it was shown that the use of low-frequency electrical stimulation of different durations (10 Hz, 8 h per day or 20 Hz, twice a day for 15 min on alternate days) during 2–3 week HU is able to attenuate or fully prevent decreases in the weight and CSA of type I fibers in rat soleus muscle [162,163]. Evidence also suggests that the application of electrical stimulation over the course of muscle reloading after a period of disuse may also be beneficial in terms of acceleration of muscle regrowth. Cotter et al. (1991) showed that low-frequency stimulation (0.3 ms pulse duration at 10 Hz for 8 h/day) allowed for accelerated recovery of rabbit soleus muscle structure and function following disuse associated with limb immobilization [164]. Mercier et al. (1999) investigated the impact of electrical stimulation (50 Hz, square waves of 0.2 ms duration, 40 min daily, 6 days a week) on soleus and extensor digitorum longus EDL muscles of young and old rats during 14-day recovery from 21-day HU [165]. This type of stimulation did not affect the rate of muscle weight recovery in rat soleus of both young and old rats but accelerated the recovery of EDL muscle mass in old rats [165]. Itoh and colleagues (2017) have recently assessed the effects of electrically evoked resistance training at different intensities (10%, 40%, 60% and 90% of maximum isometric contraction) on the recovery from muscle atrophy induced by HU [166]. Mice were subjected to 50 daily repetitions of isometric contraction exercise via electrical stimulation (5 V, 40 Hz, 2.0-ms duration, 250-ms train duration) that was applied to the lower limbs during 7-day reloading. The results showed that resistance training performed at 40% of maximum isometric contraction (3 mNm) promoted the fastest recovery of maximum isometric muscle contraction associated with marked recovery of soleus fiber CSA [166]. At 60% and 90% of maximum isometric contraction, damaged and smaller myofibers were observed [166]. The authors concluded that electrically evoked resistance training at a non-damaging intensity can facilitate the recovery of mouse soleus muscle from HU-induced atrophy [166]. The effects of microcurrent electrical nerve stimulation (MENS) on the regrowth of atrophied mouse soleus muscle were studied by Ohno and co-authors (2013) [167]. The application of MENS enhanced reloading-induced increase in soleus muscle mass and protein content which was accompanied by the upregulation of p-p70S6K and p-AKT [167]. Hence, MENS may represent an effective rehabilitation tool for atrophied and injured skeletal muscles [167]. Thus, literature analysis suggests that NMES can serve as a potential interventional strategy aimed at both mitigating skeletal muscle atrophy under disuse conditions and enhancing muscle mass recovery following a period of disuse.

Given that the parallel application of NMES with protein supplementation is able to enhance protein synthesis and attenuate disuse-related muscle atrophy [158], it is reasonable to suggest that combined intervention of non-damaging exercise (or mimetics such as NMES) and adequate nutritional support (essential amino acids (EAAs), protein) may be of benefit to the recovering skeletal muscle after a period of disuse.

### 4.3. Massage in the Form of Cyclic Compressive Loading

A recent study by Miller et al. (2018) has described the effects of massage on rat gastrocnemius muscles that were subject to cyclic compressive loading (CCL), a massage mimetic, during a period of recovery from disuse-induced atrophy [168]. CCL was applied at a 4.5 N load at 0.5 Hz frequency for 30 min every other day for four bouts during a period of 8-day reloading after 14-day HU [168]. Application of CCL during reloading significantly enhanced muscle fiber CSA by 18% compared to reloading alone, and this was accompanied by elevated protein synthesis of the myofibrillar and cytosolic, but not the mitochondrial, fractions [168]. Massage applied during the recovery of gastrocnemius also enhanced such key markers of mechanosensitive signaling as α7-integrin and phospho-FAK [168]. It is important to note that fiber CSA and protein synthesis of the myofibrillar fraction were elevated in muscle of the contralateral non-massaged limb [168]. Thus, massage in the form of CCL can be viewed as a potential anabolic intervention during skeletal muscle recovery following disuse-induced atrophy.

### 4.4. Beta2-Adrenoceptor Agonists

Beta2-adrenoceptors are the most predominant subtype of adrenoceptors in skeletal muscle, which have attracted interest as potential therapeutic targets in treatment of muscle wasting induced by both disuse and disease (please see comprehensive review by Lynch and Ryall [169]). Beta2-adrenoceptors can contribute to the regulation of muscle protein turnover and the stimulation of these receptors with selective agonists is able to increase muscle mass in mammals [169]. The effect of beta2-adrenoceptor agonists is carried out via cyclic AMP-dependent activation of PKA, Epac (exchange protein directly activated by cAMP) and ERK, which have various downstream targets controlling anabolic processes, including cyclic AMP response element binding protein (CREB), protein kinase B (AKT) and mTORC1 [169]. Beta2-adrenergic signaling can be also involved in AKT-dependent regulation of atrophy-related genes (MAFbx and MuRF1) [169]. Hostrup and co-authors (2018) have recently investigated the effect of beta2-adrenoceptor agonist (salbutamol) administration on protein turnover rates and signaling in human skeletal muscle during recovery (the first 5 h) from resistance exercise [170]. Salbutamol treatment augmented the rate of myofibrillar protein synthesis and protein breakdown as well as counteracted a negative net protein balance in human vastus lateralis after resistance exercise. Salbutamol-induced alterations in protein turnover were accompanied by a higher phosphorylation of PKA substrates, activation of AKT2, as well as modulation of mRNA expression of calpain-1, FoxO1 and myostatin [170]. In a recent rodent study, Suzuki et al. (2020) [171] demonstrated that administration of beta2-adrenoceptor agonist clenbuterol (daily 1mg/kg) during 14-day recovery from hindlimb immobilization significantly accelerated the restoration of both slow- and fast-twitch fiber CSA in rat soleus and plantaris muscles. Thus, beta2-adrenoceptor agonists appear to be a promising intervention aimed at improving skeletal muscle regrowth after disuse-induced muscle wasting. Although new generation beta2-adrenoceptor agonists (for example, formoterol) are able to elicit an anabolic response in skeletal muscle at low doses and exhibit reduced cardiovascular side effects, future studies are needed to elucidate if there could be any potential deleterious effects when beta2-adrenoceptor agonists are chronically administered.

### 4.5. Amino Acid and Protein Supplementation

Maintaining skeletal muscle mass during disuse and then restoring it during a recovery period involves processes dependent on protein turnover. First of all, a proper caloric intake can be considered as a useful strategy for attenuating muscle loss during muscle unloading as well as accelerating the restoration of muscle mass during reloading. A deficient diet that does not preserve energy balance has been shown to decrease protein synthesis by approximately 20% [172], and therefore an adequate protein intake must be provided during recovery from disuse-induced muscle atrophy. It was also shown that stimulation of muscle protein synthesis depends on the sensing of the concentration of extracellular, rather than intramuscular essential amino acids (EAAs) and EAA ingestion can stimulate the rates of synthesis of various classes of muscle proteins (myofibrillar, sarcoplasmic and mitochondrial) in a dose-dependent manner [173]. In C2C12 skeletal muscle cells, Atherton et al. (2010) showed that among EAA leucine is unique in its capacity to stimulate mTORC1 signaling [174]. Evidence suggests that EAAs, but not nonessential, are mostly responsible for the amino acid-induced stimulation of protein synthesis in human skeletal muscle, [175,176]. Branched-chain amino acids (BCCAs) such as leucine, valine, and isoleucine are known to stimulate muscle protein synthesis [177] and protect against unloading-induced muscle atrophy in rats [178]. However, in humans, intravenous infusion studies found that BCAA decreased muscle protein synthesis as well as protein breakdown, meaning a decrease in muscle protein turnover [179]. Although BCAA alone may not promote muscle anabolism in humans [179], BCAA may enhance the anabolic effect of a protein meal by increasing muscle protein synthesis in young humans [180], since the intact proteins provide all of the EAA necessary to synthesize new proteins [179].

Martin and colleagues (2013) investigated the effect of whey protein supplementation, as compared to the standard casein diet, on the recovery (7, 21 and 42 days) of skeletal muscle functional properties after a casting-induced immobilization period (8 days) in adult rats (10–11 months of age) [181]. The authors discovered that whey protein diet promoted a faster recovery of isometric force and concentric power output in plantar flexors as compared to the casein diet. Such effect could be related to the effect of whey proteins on muscle protein synthesis. The authors of this research suggest that leucine, if supplemented in a free form, is absorbed rapidly and induces its anabolic signal for protein synthesis before there is a sufficient availability of amino acids coming from dietary protein digestion. However, when leucine-rich proteins (whey proteins) are used instead of free leucine, an improvement of muscle mass recovery is observed, probably because leucine and other amino acids are elevating synchronically and generate a more sustained protein synthesis stimulation during the postprandial state [182,183].

In relation to molecular mechanisms implicated in the regulation of protein synthesis in human skeletal muscle in response to mechanical stimuli (in the form of physical exercises) and/or amino acid ingestion, it has been shown these anabolic cues may be related to the promotion of both mTORC1-dependent signaling (as assessed by phosphorylation of p70S6K and rpS6) and mTORC1-independent (rapamycin-insensitive) pathways (phosphorylation of ERK1/2, p90RSK and c-Myc expression) (reviewed in [184]).

A well-known leucine metabolite, Beta-Hydroxy-Beta-Methyl Butyrate (HMB), is an oral nutritional supplement that is known to possess pro-anabolic properties. Indeed, Wilkinson and colleagues have demonstrated that orally consumed HMB promotes acute muscle anabolism by upregulation of protein synthesis via increases in mTORC1 signaling (phosphorylation of p70S6K and rpS6) and downregulation of protein breakdown in an insulin-independent manner [185,186]. Several studies showed a positive role of HMB supplementation during muscle unloading/reloading. Deutz and co-authors (2013) found that, HMB supplementation (calcium salt, 3 g/day) is able to preserve muscle mass in healthy older adults during 10 days of bed rest [187]. A recent study showed that HMB administration (340 mg/kg body weight) is able to inhibit HU-induced increase in MuRF1 and MAFbx expression and partially prevent soleus muscle atrophy in mice [188]. Alway and colleagues (2013) demonstrated a beneficial effect of HMB on plantaris muscle of 34-month-old rats [189]. The animals daily received Ca–HMB (340 mg/kg body weight) throughout 14-day HU and subsequent 14-day recovery. After reloading, plantaris muscle mass and fiber CSA were significantly greater in HMB-treated rats relative to vehicle controls [189]. This effect was accompanied by enhanced proliferation of satellite cells and increased nuclear protein abundance of proliferation markers [189]. Furthermore, treatment with HMB resulted in increased AKT phosphorylation; however, phosphorylation levels of mTORC1, P70S6K and 4E-BP1 remained unaltered [189]. In aged rats, HMB treatment during unloading and reloading also led to increased plantaris muscle mass after reloading as well as partial preservation of fiber atrophy in both slow soleus and fast plantaris muscles in response to HU [190]. The ability of HMB to preserve skeletal muscle loss could be associated with inhibition of myonuclear apoptosis via regulation of mitochondrial-associated caspase signaling [190].

Nakanishi et al. (2016) hypothesized that nucleoprotein (i.e., a combination of amino acids and nucleotides) supplementation would enhance the recovery of muscle mass to a greater extent than reloading alone after a period of unloading [191]. Nucleoprotein was extracted from salmon soft roe and administered to rats of the 5-day reloading group via a catheter twice per day at a dose of approximately 800 mg/kg. Compared to the reloading group without nucleoprotein supplementation, nucleoprotein-treated reloaded rats had larger soleus muscles and fiber CSA, higher levels of phosphorylated rpS6, as well as higher amount of myonuclei and myogenin-positive cells [191]. Thus, nucleoprotein supplement may represent a possible intervention strategy aimed at recovering skeletal muscle mass following disuse conditions such as bedrest and cast immobilization.

### 4.6. Creatine Supplementation

Although there are numerous speculations that creatine supplementation could lead to an increase in lean body mass in active individuals, Backx et al. (2017) [192] have recently demonstrated that creatine administration failed to preserve skeletal muscle mass or strength during leg immobilization in healthy, young males. In this study, healthy young men (*n* = 30; aged 23 ± 1 years) were randomly assigned to either a creatine or a placebo group. Subjects received placebo or creatine supplements for 5 days before (20 g/day) and during 7-day leg immobilization as well as during subsequent 7-day recovery (5 g/day) from leg immobilization. Creatine supplementation failed to accelerate the restoration of the CSA of quadriceps muscle or enhance leg strength regain after 1 week of recovery period [192]. However, Hespel et al. (2001) earlier showed greater quadriceps muscle regain following a period of disuse (2-week leg immobilization) when creatine was supplemented during a more prolonged period of recovery including a 10-week resistance-type exercise training program [193]. It is worth noting that the anabolic effect of creatine supplementation was evident for both fast- and slow-twitch muscle fibers. Creatine intake hypertrophied type I, type IIa and type IIb fibers in human vastus lateralis muscle to a similar degree during the 10-week rehabilitative knee-extension training after a period of disuse [193]. This finding suggests that muscle fiber distribution is probably not critical to the impact of creatine supplementation on muscle mass during training-based recovery. It is important to note that creatine supplementation for 10 weeks during rehabilitation increased the content of myogenic regulatory factor 4 (MRF-4). Interestingly, the change in MRF4 protein content from day 0 to 10 weeks of rehabilitation correlated with the concomitant change in average myofiber CSA. Thus, creatine supplementation may be of benefit in support of skeletal muscle mass and strength regain during more prolonged active rehabilitation.

### 4.7. Antioxidant and Anti-Inflammatory Supplementation

Protein synthesis in skeletal muscle can be influenced by oxidative stress and inflammation, which are known to be associated with disuse-induced atrophy [194]. Since both factors may trigger increased proteolysis during periods of prolonged disuse [80,195], antioxidant/anti-inflammatory supplementation might serve as a possible countermeasure to prevent protein loss.

Antioxidants, such as resveratrol (3,5,4′-trihydroxystilbene), has been shown to reduce oxidative stress, restore mitochondrial function, and promote myogenesis and hypertrophy in vitro [196]. Bennett et al. (2013) tested the hypothesis that resveratrol supplementation would improve muscle regeneration in muscles of aged rats following muscle disuse [197]. Fisher 344 × Brown Norway rats (32 months of age) were subjected to 14-day HU followed by 14-day reloading. Resveratrol was administered via oral gavage at a dose of 125 mg/kg/day throughout the study. Resveratrol supplementation was unable to attenuate a decrease in plantaris muscle weight during HU but it improved muscle mass during reloading after HU [197]. Moreover, resveratrol-treated rats demonstrated an enhanced reloading-induced increase in CSA of type IIA and IIB muscle fibers. As muscles from aged animals display an impaired recovery following disuse-induced muscle atrophy, the results obtained by Bennett et al. (2013) may be relevant for clinical geriatric practice associated with the age-associated loss of muscle mass and function (sarcopenia) [197]. These positive changes could be related to the suppression of pro-apoptotic signaling. Indeed, during the recovery period, resveratrol suppressed both cleaved caspase 3 and cleaved caspase 9 in the plantaris muscles relative to the vehicle-treated group [197]. In the recovery group, resveratrol administration also elevated AMPK and phospho-AMPK protein abundance in the plantaris muscle [197].

Tea polyphenols are traditionally regarded as substances with antioxidant, antidiabetic, and anti-inflammatory properties [198,199,200]. Active substances extracted from the leaves of the *Camellia sinensis* plant include epigallocatechin gallate, epicatechin gallate, gallocatechin, and epigallocatechin [201]. Ota et al. (2011) have shown that ingestion of tea catechins significantly inhibited HU-induced decrease in force in isolated murine soleus muscle as well as suppressed oxidative modification of myofibrillar proteins [202]. In two separate studies involving aged rats, Alway and co-workers have tested the effects of epigallocatechin-3-gallate (EGCg) and green tea extract (GTE) on slow-twitch soleus and fast-twitch plantaris muscles after 14-day unloading and subsequent 14-day reloading [203,204]. It was shown that EGCg did not prevent unloading-induced atrophy in both muscles, however, it improved plantaris muscle recovery (but not soleus) after the atrophic stimulus [203]. GTE increased satellite cell proliferation and differentiation in plantaris and soleus muscles during recovery from HU compared with vehicle-treated muscles and decreased oxidative stress and abundance of the Bcl-2-associated X protein yet failed to improve muscle recovery in reloaded muscles [204]. This highlights the fact that, although satellite cells are important in muscle repair, simply having more satellite cells or their daughter cells does not guarantee improved muscle recovery, at least in old rats [204]. Recently, Aoki et al. (2019) have investigated the effect of polyphenol-rich fraction of black tea (named E80) on mouse soleus muscle during 2-week hindlimb unloading and subsequent 5- and 10-day reloading [205]. The authors have demonstrated that E80 administration did not prevent soleus muscle atrophy during unloading, however, E80 accelerated the restoration of soleus muscle mass and fiber CSA after 5 and 10 days of reloading [205]. This E80-induced improved recovery of the mouse soleus muscle was accompanied by a significant increase in the phosphorylation levels of AKT, p70S6K and rpS6 after 5-day reloading suggesting activation of mTORC1 signaling [205].

Curcumin (diferuloylmethane) is a component of the spice turmeric (*Curcuma longa*), which exhibits anti-inflammatory and antioxidant properties [206]. Curcumin was shown to prevent the induction of signaling pathways (i.e., NF-κB, xanthine oxidase, β-catenin) which are usually activated during unloading/disuse and associated with the induction of the UPS and the apoptotic pathway [207]. In a study by Vazeille et al. (2012), adult rats (8 months old) were daily treated with curcumin (1 mg/kg) or vehicle during hindlimb immobilization (8 days) and subsequent recovery (10 days) [207]. It was revealed in gastrocnemius muscle that during 10-day recovery, curcumin treatment induced an increase in X-linked inhibitor of apoptosis protein (XIAP) content as well as blocked the elevated proteasome chymotrypsin-like and apoptosome-linked caspase-9 activities, and thereby significantly improved the recovery of muscle mass and fiber CSA [207].

**Figure 4:**
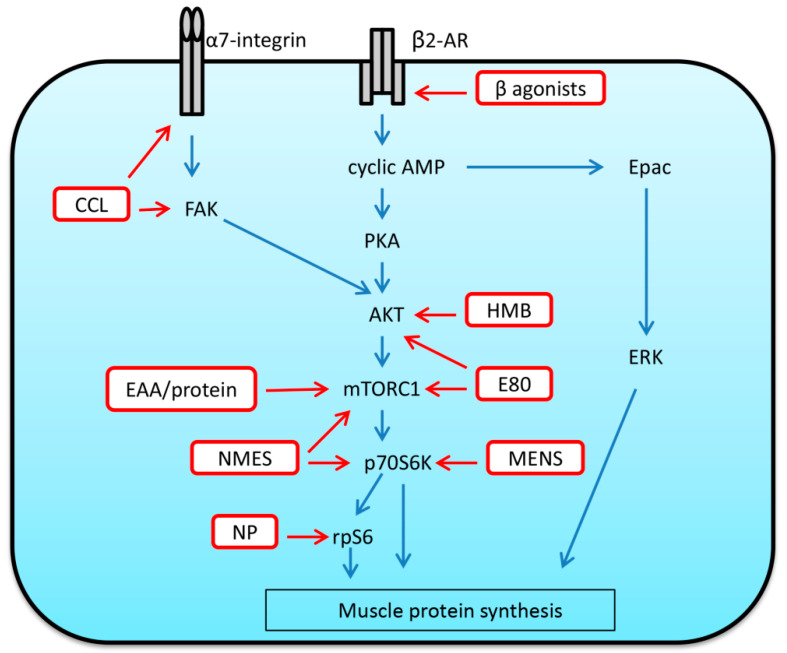
Schematic illustration of the effects of possible therapeutic interventions on the anabolic pathways in reloaded skeletal muscle. Arrows indicate stimulatory signals. β2-AR—beta 2 adrenoreceptor, PKA—protein kinase 2, mTORC1—mammalian/mechanistic target of rapamycin complex 1, p70S6K—ribosomal protein S6 kinase p70, rpS6—ribosomal protein S6, FAK—focal adhesion kinase, Epac—exchange protein directly activated by cyclic AMP, ERK—extracellular signal-regulated kinase, AKT—protein kinase B, CCL—cyclic compressive loading, HMB—beta-hydroxy-beta-methyl butyrate, EAA—essential amino acids, NMES—neuromuscular electrical stimulation, MENS—microcurrent electrical nerve stimulation, E80—polyphenol-rich fraction of black tea, NP—nucleoprotein (a combination of amino acids and nucleotides).

An illustration of the effects of the above-described interventions on the anabolic signaling pathways is provided in Figure 4.

### 4.8. Putative Molecular Therapeutic Targets and Future Directions

As discussed above, skeletal muscle reloading after a period of disuse/unloading requires the activation of both anabolic and catabolic signaling pathways leading to increases in the rates of protein synthesis and degradation with much more pronounced protein synthetic response relative to proteolysis resulting in a complete recovery of muscle mass. Rodent studies using pharmacological approaches or genetic models have uncovered a number of molecules that may play important roles in the activation/enhancement of muscle protein synthesis during recovery from mechanical unloading/disuse. These molecules might represent possible targets for therapeutic interventions aimed at improving protein synthesis/turnover and thereby accelerating skeletal muscle regrowth following disuse atrophy (Table 3).

Although a lot of anabolism-related signaling events occurring in skeletal muscle during reloading have been established, there are mechanisms that still remain uncovered. For example, future studies should focus on protein phosphatase 2A, an enzyme which may play a key role in dephosphorylating such important anabolic molecules as p70S6K, 4E-BP1 (mTORC1 targets), eEF2 and others. In comparison to signaling events implicated in the regulation of translational efficiency (initiation and elongation of mRNA translation), ribosomal biogenesis-related signaling (regulation of c-Myc, UBF, etc.) determining capacity for protein synthesis is yet to be investigated in both rodent and human skeletal muscle during reloading. Furthermore, while the contribution of IGF-1/AKT/mTORC1, AKT/GSK3β and ERK/p90RSK signaling pathways to the regulation/activation of protein synthesis during muscle reloading is relatively well studied, accumulating evidence suggests that other pathways such as Wnt/βcatenin, Hippo/YAP, DGKζ/PA/mTORC1 may impact muscle protein synthesis in reloaded skeletal muscle. Future research should focus on these signaling cascades in order to provide molecular-level insights into the regulation of muscle protein synthesis during reloading after disuse-induced muscle atrophy.

As the magnitude and duration of changes in the rates of muscle protein synthesis in response to unloading/reloading are muscle specific [105,208], future investigations should take into account both muscle function (extensors/flexors) and distribution of fiber types (predominantly slow/fast or mixed). This point is corroborated by a recent study demonstrating that the content of the key anabolic markers can differ between fiber types in human vastus lateralis muscle (protein levels of p70S6K and eEF2 were significantly higher in type II than type I fibers) [209]. It is also worth noting that in order to obtain a full picture of molecular events (mechanisms) in a specific muscle, it is important to collect samples at multiple time points over the course of unloading/reloading.

## 5. Conclusions

Over the past decade considerable progress has been made in our comprehending the molecular mechanisms underlying the recovery of skeletal muscle mass following a period of disuse/mechanical unloading. A better understanding of the mechanisms of protein turnover may ultimately lead to development of therapies that can enhance skeletal muscle recovery from disuse-induced atrophy. Multiple signaling pathways controlling both protein synthesis and protein degradation appear to be involved in the restoration of muscle protein content during reloading after mechanical unloading. Evidence suggests that an important role in the regulation of protein synthesis in reloaded skeletal muscle belongs to such molecules as AKT, mTOR, GSK-3β, SAC, NO and AMPK. Activation of proteolysis at the early stages of reloading is also important as it apparently helps to eliminate or replace damaged or atrophy-induced proteins. Possible strategies aimed at enhancing skeletal muscle regrowth following disuse atrophy may include both physical activity interventions (low-impact exercises, massage, NMES) and dietary supplementation (e.g., whey protein, nucleoprotein, resveratrol, curcumin).

## Figures and Tables

**Table 1:** The effect of reloading after mechanical unloading on the key anabolic markers in rodent soleus muscle.

Animal	Reloading Duration	Parameters	References
rat	18 h, 7 days	Protein synthesis ↑	[116]
rat	3 and 7 days	Protein synthesis ↑	[105]
rat	12 h, 24 h	Protein synthesis ↑	[117]
rat	2 and 4 days	Total RNA ↑	[119]
rat	6 h, 12 h and 24 h	c-Myc mRNA ↑	[117]
rat	12 h	p-p70S6K (Thr389) ↑ p-rpS6 (Ser240/244) ↑ p-4E-BP1 (Thr36/46) ↑	[117]
rat	3 days	p-AKT (Thr473) ↑ p-p70S6K (Thr389) ↑	[134]
rat	3 days	p-p70S6K (Thr389) ↑ GSK3β (Ser9) ↑	[127]
rat	3 days	p-p70S6K (Thr389) ↑ p-rpS6 (Ser240/244) ↑	[135]
rat	3 days	p-p70S6K (Thr389) ↑ p-4E-BP1 (Thr36/46) ↑ GSK-3β (Ser9) ↑	[105]
rat	5 h, 24 h, 14 days	ERK1/2 activity ↑	[137]
rat	3 days	Total eIF2B ↑	[104]
rat	14 days	p-AKT (Thr473) **─** p-p70S6K (Thr389) ─ GSK3β (Ser9) ─	[136]
rat	7 and 14 days	GSK3β (Ser9) ─	[104]
mouse	7 days	Total RNA	[139]
mouse	3 days	p-AKT (Thr473) ↑ GSK3β (Ser9) ↑	[106]
mouse	1 and 3 days	p-AKT (Thr473) ↑ p-p70S6K (Thr389) ↑	[138]

↑ indicates a significant increase compared to cage control animals; **─** indicates no change compared to control animals.

**Table 2:** The effect of reloading after mechanical unloading on the markers of proteolysis and inflammation in rodent soleus muscle.

Animal	Reloading Duration	Parameters	References
rat	18 h	Protein degradation ↑ Ub-protein conjugates ↑ mRNA levels of C8 and C9 proteasome subunits) ↑ Ub mRNA levels ↑ Calpain-2 mRNA levels↑	[116]
rat	7 days	Protein degradation **─** Ub-protein conjugates **─** mRNA levels of C8 and C9 proteasome subunits) **─** Ub mRNA levels **─** Calpain-2 mRNA levels **─**	[116]
rat	3 days	Ub-protein conjugates ↑ Proteasome activity ↑	[105]
rat	12 h, 24 h	Total calpain activities ↑	[20]
rat	3 days	MuRF-1 and MAFbx mRNA expression ↑	[111]
rat	7 days	MuRF-1 and MAFbx mRNA expression **─**	[111]
rat	1 and 5 days	MuRF-1 mRNA expression **─**	[109]
rat	5 days	Beclin-1 ↑	[109]
rat	1 and 5 days	Calpain-1 mRNA expression **─**	[109]
rat	1 day	Caspase-3,-8,-9 ↑	[109]
rat	1 and 5 days	TNFα ↑	[109]
rat	24 h	interleukin-6 ↑ interleukin-1β ↑	[109]
mouse	4 days	CD 11b expression↑ CD 11c expression↑ CD68^+^ cells ↑	[144]
mouse	3 days	Macrophage and neutrophil concentrations ↑	[148]
mouse	2 and 4 days	Macrophage concentrations ↑	[147]
mouse	24 h	Ub expression ↑	[139]
mouse	2 days	Ub-protein conjugates ↑ Calpain-3 content ↑	[78]

↑ indicates a significant increase compared to cage control animals; **─** indicates no change compared to control animals.

**Table 3:** Molecules that were shown to play an important role in the regulation of protein synthesis/turnover during muscle reloading and which may serve as potential therapeutic targets.

Potential Therapeutic Targets	Possible Function during Muscle Reloading	References
SAC (possibly TRPC1)	activates mTORC1 signaling and protein synthesis during acute reloading	[117]
Beta2-adrenoreceptor	beta2-adrenoceptor agonists enhance protein synthesis via cyclic AMP/PKA signaling	[171]
nNOS/NO	activates mTORC1 signaling and protein synthesis	[124]
IFG-1	activates PI3K/AKT/mTORC1 pathways and protein synthesis	[122,123]
PI3K/AKT	activates protein synthesis	[127]
mTORC1	activates protein synthesis	[126]
AMPK	negatively regulates protein synthesis attenuating muscle regrowth	[128]
GSK3β	negatively regulates protein synthesis attenuating muscle regrowth	[129]
Calpain-3	supports protein turnover during muscle reloading	[78,144]

## Abbreviations

40S	small ribosomal subunit
4E-BP1	eukaryotic initiation factor 4E binding protein
60S	large ribosomal subunit
AKT	protein kinase B
AMPK	AMP-activated protein kinase
AMPK-DN	transgenic mice specifically overexpressing dominant-negative AMPK α1 subunit in skeletal muscle
BCCA	branched-chain amino acids
CCL	cyclic compressive loading
cGMP	cyclic guanosine monophosphate
c-Myc	c-myelocytomatosis oncogene
CREB	cyclic AMP response element binding protein
CSA	cross-sectional area
DGKζ	zeta isoform of diacylglycerol kinase
Dvl	disheveled protein
EAA	essential amino acids
EDL	extensor digitorum longus
eEF2	eukaryotic elongation factor 2
eEF2K	eukaryotic elongation factor 2 kinase
EGCg	epigallocatechin-3-gallate
eIF2B	eukaryotic initiation factor 2B
eIF3f	eukaryotic initiation factor 3f
eIF4E	eukaryotic initiation factor 4E
eIF4G	eukaryotic initiation factor 4G
Epac	exchange protein directly activated by cyclic AMP
ERK	extracellular signal-regulated kinase
FAK	focal adhesion kinase
FoxO	forkhead box O protein
FRZ	frizzled protein (receptor)
GSK-3β	glycogen synthase kinase-3β
GSK-3β KO	mice lacking muscle GSK-3β
HMB	beta-hydroxy-beta-methyl butyrate
HSP70	70 kDa heat shock protein
HU	hindlimb unloading
IGF-1	insulin-like growth factor 1
IL-6	interleukin-6
IRS-1	insulin receptor substrate 1
LATS	large tumor suppressor kinase
LC3	microtubule-associated proteins 1A/1B light chain 3B
L-NAME	N(gamma)-nitro-L-arginine methyl ester
MAFbx	muscle atrophy F-box protein/atrogin-1
MAPK	mitogen-activated protein kinase
MEK	mitogen-activated protein kinase kinase
MGF	mechano-dependent growth factor
MRF-4	myogenic regulatory factor 4
MENS	microcurrent electrical nerve stimulation
mTORC1	mammalian/mechanistic target of rapamycin complex 1
MuRF1	muscle RING finger protein
NF-κB	nuclear factor kappa B
nNOS	neuronal NO synthase
NO	nitric oxide
NMES	neuromuscular electrical stimulation
p70S6K	ribosomal protein S6 kinase p70
p90RSK	ribosomal protein S6 kinase p90
PA	phosphatidic acid
PBS	phosphate-buffered saline
PI3K	phosphatidylinositol 3-kinase
PKA	protein kinase A
Pol	polymerase
rDNA	ribosomal DNA
REDD1	regulated in development and DNA damage response 1 protein
Rheb	Ras homolog enriched in brain
ROS	reactive oxygen species
RP	ribosomal proteins
rpS6	ribosomal protein S6
rRNA	ribosomal RNA
SAC	stretch-activated ion channels
siRNA	small interfering RNA
SL-1	selective factor 1
TAZ	transcriptional coactivator with PDZ-binding motif
TGF	transforming growth factor
TNF	tumor necrosis factor
TRIM	1-(2-trifluoromethyl-phenyl)-imidazole
TRPC1	transient receptor potential canonical 1 channel
TRPV1	transient receptor potential cation channel subfamily V member 1
TSC2	tuberous sclerosis complex 2
TWEAK	TNF-like weak inducer of apoptosis
Ub	ubiquitin
UBF	upstream binding factor 1
UBR5	E3 ubiquitin ligase
ULK1	unc-51-like autophagy activating kinase
UPS	ubiquitin–proteasome system
YAP	Yes-associated protein
